# Limits to the “Medical Clearance” of Patients Presenting With Psychiatric Changes: A Case Report and Review of the Literature

**DOI:** 10.1155/crps/7070787

**Published:** 2026-07-02

**Authors:** Jordan Pelc, Gregory Chandler, Riley Rose

**Affiliations:** ^1^ Division of Hospital Medicine, Sinai Health System, Toronto, Ontario, Canada, sinai.org; ^2^ Department of Family and Community Medicine, Temerty Faculty of Medicine, University of Toronto, Toronto, Ontario, Canada, utoronto.ca; ^3^ Department of Psychiatry, Sinai Health System, Toronto, Ontario, Canada, sinai.org; ^4^ Department of Psychiatry, Temerty Faculty of Medicine, University of Toronto, Toronto, Ontario, Canada, utoronto.ca; ^5^ Department of Psychiatry, Vancouver General Hospital, Vancouver, British Columbia, Canada, vch.ca; ^6^ Department of Psychiatry, University of British Columbia, Vancouver, British Columbia, Canada, ubc.ca

## Abstract

We discuss a case of a 54‐year‐old woman with a history of schizoaffective disorder and Parkinson’s disease who presented with altered mental status, including weakness, falls, medication refusal, and paranoia, heralding medical deterioration. The treating teams initially suspected that this presentation was medically driven, and she had multiple admissions to general medicine to look for an “organic” explanation of her clinical status. Despite this, no medical driver was identified, and she was diagnosed with progression of her primary psychiatric disease. After she was “medically cleared” and was admitted to the inpatient psychiatry unit for 10 days, she was found to be medically unstable, with progressive dysphagia and decreased responsiveness. She was then admitted to the intensive care unit (ICU), where was found to have dysphagia, aspiration pneumonia, lung abscess, severe hypernatremia, and associated diabetes insipidus, and ultimately died of her medical illness. This case illustrates important limitations of the “medical clearance” of patients with psychiatric presentations, including limited literature and clinical guidance in this area, and risks of the term “medical clearance” itself, which may inappropriately discourage medical reassessments when indicated. We review practices that can help reduce these risks.

## 1. Background

It is well known that psychiatric presentations can be driven by medical illness and that diagnostic criteria for psychiatric diseases commonly require ruling out a general medical disorder. Despite this, there is little literature on how to rule out medical illnesses in psychiatric presentations. This process, frequently referred to as “medical clearance,” has received little study outside of the emergency department. Both general medical physicians and psychiatrists frequently encounter undifferentiated illness, including undifferentiated behavioral presentations, and have to make clinical decisions about their workup. This case illustrates best practice approaches to “medical clearance” as well as some challenges with this process.

## 2. Description of Case

A 54‐year‐old woman from long‐term care presented to the emergency department with a 1‐week history of weakness, falls, medication refusal, and paranoia. Her past medical history included schizoaffective disorder and Parkinson’s disease, as well as transient ischemic attack (TIA) and dyslipidemia. Her medications included levodopa/carbidopa, quetiapine, benztropine, and senokot, and there had been no recent medication changes. At baseline, she had cognitive impairment and was dependent on basic activities of daily living but could mobilize independently and was cooperative with care.

In the emergency department, her physical examination was remarkable for agitation and was otherwise unrevealing. Vital signs were within normal limits. Investigations, including complete blood count, electrolytes, glucose, creatinine, calcium, magnesium, thyroid‐stimulating hormone, vitamin B12 level, and chest X‐ray, were within normal limits. She was admitted to the general internal medicine (GIM) service to rule out a new medical illness driving her mental status changes.

Upon admission to the GIM unit, her repeat sodium level was found to be high (153 mmol/L). This was felt to be related to decreased fluid intake when being moved from the ED to the inpatient ward. This resolved with intravenous fluids and remained within normal limits throughout the rest of her admission without intravenous hydration. Importantly, her sodium had been within normal limits on initial presentation to the ED, and her behavioral disturbances were present prior to the finding of hypernatremia. While admitted, blood cultures came back negative, and CT brain demonstrated no new changes. However, her behavioral changes, including medication refusal and paranoia, persisted. Given that no new medical illness was discovered, she was “medically cleared” and referred to the consultation‐liaison (CL) psychiatry team. They suggested an increase of her quetiapine dose and consideration of either electroconvulsive therapy or clozapine, though the underlying etiology of her deterioration was felt to be unclear. She was discharged back to long‐term care 5 days following her admission.

One day following discharge, she again presented to the emergency department. Her long‐term care facility reported that she had been aggressive toward staff, resisting help, and refusing medication. In the 24 h since discharge from hospital, she suffered three falls. She was therefore readmitted from the emergency department to GIM. She again appeared paranoid and resistant to medications, though her vital signs were within limits, and there were no new focal medical findings on physical examination. Repeat bloodwork was unrevealing, including normal sodium and creatinine. A repeat CT head was unchanged. Despite efforts, no medical cause for her presentation was found.

The CL psychiatry team was again consulted. Given multiple negative medical workups, it was felt that her deterioration was likely consistent with the worsening of her primary psychiatric illness. The medical team therefore did not continue to order new investigations, and the CL team advocated for transfer to the hospital’s psychiatry unit for stabilization of psychotic symptoms.

Three days into her admission on the psychiatry unit, the nursing staff noted that she appeared to be having swallowing difficulties. The speech‐language pathologist subsequently assessed her and arranged for her to be on a dysphagia diet. Ten days into her psychiatry admission, the nursing team reported evidence of physical deterioration, including decreased oral intake, less responsive behavior, and unintelligible speech. The inpatient psychiatry team consulted the GIM consult team, who recommended basic bloodwork and agreed to see the patient later that day. However, within a few hours of requesting the GIM consultation, the patient became hypotensive and tachypneic. The GIM consult team was called back and recommended an urgent chest X‐ray, which did demonstrate consolidation. The patient’s bloodwork returned after the X‐ray was ordered and showed a critically high sodium of 172. She was urgently transferred to the hospital’s intensive care unit (ICU).

Over a 3‐month period, the patient was transferred between the ICU and GIM several times. She was diagnosed with aspiration pneumonia, pulmonary abscess, and both central and nephrogenic diabetes insipidus. No unifying diagnosis was ever elucidated. She continued to require aggressive intravenous fluids. It ultimately became clear that intravenous hydration was not consistent with her goals of care, and fluids were discontinued. She died in the hospital shortly thereafter.

## 3. Discussion and Conclusion

Medical illness can manifest as a broad range of psychiatric presentations, including disorders of mood, anxiety, psychosis, cognition, and personality [1]. There are scattered case reports in this area [2] but little organized literature. The proportion of psychiatric presentations caused or exacerbated by medical illness has been cited as anywhere from 19%–80%, though the reliability of these figures is limited as they depend on the range of medical diagnoses considered and the reliability of the associated diagnostics [3]. Psychiatric diagnosis in general requires ruling out the contribution of a general medical condition, but there is no gold standard for doing so [3]. There is no medical specialty responsible for ruling out medical drivers of psychiatric illness, and this task often falls to either generalist physicians or psychiatrists.

One of the few areas for which there is literature in this area is in the emergency department, where the term “medical clearance” is commonly used. There is disagreement between emergency medicine and psychiatry guidelines regarding the extent of workup required to deem a patient “medically clear” [4]. In 2017, a task force of the American Association for Emergency Psychiatry (AAEP) developed consensus recommendations for the medical evaluation of psychiatric patients presenting to United States emergency departments [5]. It highlighted that there are currently no randomized clinical trials comparing different strategies for medically assessing psychiatric patients in the ED, nor are there randomized trials investigating reliable markers of medical illness in psychiatric patients. The best available evidence is from retrospective studies. These indicate that most missed medical illnesses in this population could have been identified with more complete bedside assessments rather than more investigations. Based on these studies, the AAEP concluded that at minimum, patients presenting with psychiatric symptoms to the emergency department require a thorough history and physical examination, including past medical and psychiatric history, review of systems, substance use history, physical examination including vital signs, and mental status examination, ideally including orientation and cognition. Features likely warranting further medical workup included new onset psychiatric symptoms below the age of 12 or above the age of 45, patients aged 65 years or older, cognitive deficits or delirium, positive review of systems indicative of medical etiology, focal neurologic findings or evidence of head injury, substance intoxication, withdrawal or exposure, decreased level of awareness, and other pertinent positives on history and physical. Subsequent studies have corroborated the recommendations made by the AAEP, including the focus on bedside assessment with little added value from routine broad investigations [6–9].

Importantly, the AAEP emphasizes that the term “medical clearance” should not be used, as the term can have different meanings to different clinicians and therefore can be associated with dangerous miscommunications [4]. Because new medical problems may manifest at a later time, a patient should never be considered “medically cleared.” Rather, a transfer note should accompany a patient after a medical evaluation, indicating that the patient is medically stable and appropriate for treatment in a psychiatric setting. By this, they mean that the patient’s behavioral disturbance at the time of their medical evaluation is felt to be unlikely due to a medical condition, and the patient’s current medical needs can be met within the capabilities of the receiving psychiatric facility [5] (e.g., frequent vital signs monitoring or IV medications may not be within the scope of a psychiatric care environment).

To our knowledge, there are no other reported cases of patients admitted to an inpatient psychiatry unit directly from a medical unit where they were “medically cleared” who then died of medical illness on the same admission. This case illustrates key limitations of “medical clearance.” First, it is imprecise. Unlike specific conditions which can be excluded by showing that a patient does not meet diagnostic criteria, there are no specific criteria for ruling out the contribution of any possible medical condition to a psychiatric presentation. There is no algorithm for “medical clearance.” In our case, while on both GIM admissions, a history, physical examination, and set of investigations were all used to demonstrate that there was no noted active medical process which explained the patient’s mental status changes, it would be false to say that medical illness was formally ruled out. Rather, no contributing medical illness was identified, but the possibility of a medical process that was not yet evident remained.

Second, “medical clearance” can be limited by the evaluation of a single moment in time. While it may be true that a patient was medically stable or did not have manifest evidence of a medical condition driving their psychiatric presentation at the time that they were evaluated, presentations can evolve, and the same may not be true on subsequent evaluations. A patient can be deemed “medically stable” cross‐sectionally in time but never “medically cleared.” In our case, the patient’s sodium was found to be within normal limits on multiple occasions, only to be critically elevated subsequently.

Third, and relatedly, the term “medical clearance” is itself limiting: it risks biasing clinicians against appreciating new evidence of medical illness if the patient’s status changes. In our case, the patient did show signs of medical deterioration that might have been appreciated earlier in her hospital course had she been viewed as potentially medically active. For instance, well prior to her admission to the ICU with a critically elevated sodium, our patient did briefly have a more moderately elevated sodium on her first GIM admission. This had normalized with intravenous fluids. The assumption was that this was a self‐limited anomaly, but in hindsight, monitoring for the possibility that this might recur would have afforded earlier identification of her subsequent clinical deterioration. Further, our patient did show signs of clinical deterioration throughout her stay on the psychiatry floor, as evidenced by the involvement of the speech‐language pathologist to evaluate swallowing and order a dysphagia diet. However, she was only seen by GIM 10 days into her admission on that floor. The thinking seems to have been that she had already been “medically cleared” and therefore, evidence of deterioration was not interpreted as such. We wonder if a more dynamic assessment of medical stability may have avoided an ICU transfer and facilitated a more peaceful palliative plan.

Finally, it should be noted that one solution to dichotomized medical vs. psychiatric teams is joint medical‐psychiatry units, where patients can get access as needed to both kinds of care. These have been shown to provide higher quality care to complex patients but are limited by difficulties maintaining adequate staffing with both sets of competencies and by unclear cost‐benefit [10–12].

In summary, there are significant limitations to ruling out the contribution of medical drivers to seemingly psychiatric presentations. There are a number of practices that are recommended to best manage these limitations, namely, (1) ensure complete bedside assessments (rather than routine investigations) with thorough histories including past medical and psychiatric history, review of systems, and substance use history, physical examination including vital signs, and mental status examination, ideally including orientation and cognition. (2) Explore further medical workup under any of the following circumstances: new onset psychiatric symptoms below the age of 12 or above the age of 45; patients aged 65 years or older; cognitive deficits or delirium; positive review of systems indicative of medical etiology; focal neurologic findings or evidence of head injury; substance intoxication, withdrawal, or exposure; decreased level of awareness; and other pertinent positives on history and physical. (3) Do not use the term “medical clearance” but instead determine medical stability for treatment in a psychiatric setting; recognize that new evidence of medical issues may emerge. (4) Continue to evaluate medical drivers of psychiatric illness, in particular for patients who deteriorate or do not improve.

## Funding

No funding was received for this manuscript.

## Consent

No written consent has been obtained from the patient as there is no patient‐identifiable data included in this case report. Nonessential patient demographics were changed.

## Conflicts of Interest

The authors declare no conflicts of interest.

## Data Availability

The data that support the findings of this study are available upon request from the corresponding author. The data are not publicly available due to privacy or ethical restrictions.
